# The salivary microbiota as a diagnostic indicator of oral cancer: A descriptive, non-randomized study of cancer-free and oral squamous cell carcinoma subjects

**DOI:** 10.1186/1479-5876-3-27

**Published:** 2005-07-07

**Authors:** DL Mager, AD Haffajee, PM Devlin, CM Norris, MR Posner, JM Goodson

**Affiliations:** 1 The Forsyth Institute, 140 The Fenway, Boston, MA, USA; 2 Brigham and Women's Hospital, 27 Francis Street, Boston, MA, USA; 3 Dana Farber Cancer Institute, 44 Binney Street, Boston, MA, USA

**Keywords:** Oral Squamous Cell Carcinoma, Oral mucosa, bacterial markers, bacteria, early detection

## Abstract

**Background:**

The purpose of the present investigation was to determine if the salivary counts of 40 common oral bacteria in subjects with an oral squamous cell carcinoma (OSCC) lesion would differ from those found in cancer-free (OSCC-free) controls.

**Methods:**

Unstimulated saliva samples were collected from 229 OSCC-free and 45 OSCC subjects and evaluated for their content of 40 common oral bacteria using checkerboard DNA-DNA hybridization. DNA counts per ml saliva were determined for each species, averaged across subjects in the 2 subject groups, and significance of differences between groups determined using the Mann-Whitney test and adjusted for multiple comparisons. Diagnostic sensitivity and specificity in detection of OSCC by levels of salivary organisms were computed and comparisons made separately between a non-matched group of 45 OSCC subjects and 229 controls and a group of 45 OSCC subjects and 45 controls matched by age, gender and smoking history.

**Results:**

Counts of 3 of the 40 species tested, *Capnocytophaga gingivalis*, *Prevotella melaninogenica *and *Streptococcus mitis*, were elevated in the saliva of individuals with OSCC (p < 0.001). When tested as diagnostic markers the 3 species were found to predict 80% of cancer cases (sensitivity) while excluding 83% of controls (specificity) in the non-matched group. Diagnostic sensitivity and specificity in the matched group were 80% and 82% respectively.

**Conclusion:**

High salivary counts of *C. gingivalis*, *P. melaninogenica *and *S. mitis *may be diagnostic indicators of OSCC.

## Background

Each year nearly 30,000 Americans are diagnosed with oral cancer. 90% of these lesions are oral squamous cell carcinomas [1]. Despite advances in surgery, radiation and chemotherapy, the five-year survival rate is 54%, one of the lowest of the major cancer sites, and this rate has not improved significantly in recent decades [2-4]. Worldwide, the problem is much greater, with over 350,000 to 400,000 new cases being found each year [5]. The disease kills one person every hour – more people than cancers of the cervix, brain, ovary, testes, liver, kidney, malignant melanoma or Hodgkin's lymphoma [5,6]. In the United States, African American males suffer the highest incidence and lowest survival rates of any group. From 1985 to 1996, the five-year survival rate for tongue carcinoma in African-American men was 27%, compared with a 47% five-year survival rate among white men [7]. In 2001, similar five-year survival rates were found in a study of oral and pharyngeal cancer among African-American and White men [8]. Notably, incidence in young adults (<40 years) is increasing in the U.S. [9,10] and worldwide [11,12].

Early detection followed by appropriate treatment, can increase cure rates to 80 or 90%, and greatly improve the quality of life by minimizing extensive, debilitating treatments [5,13]. Despite the accessibility of the oral cavity to direct examination, these malignancies are often not detected until a late stage [5,14,15]. Oral cancer is unusual in that it carries a high risk of second primary tumors. Patients who survive a first cancer of the oral cavity have up to a 20-fold increased risk of developing a second primary oral cancer and that risk lasts 5–10 years and sometimes longer [16].

Major risk factors for oral cancers in the United States are use of tobacco and alcohol, which account for 75 to 80% of all oral cancers [5,17]. Although tobacco is a well-recognized risk factor for OSCC, the public is generally unaware that alcohol synergizes with tobacco. Those who both smoke and drink have 15 times the risk of developing oral cancer [5]. Notably, some oral cancer patients have no known risk factors, and the disease in this population may pursue a particularly aggressive course [18].

The American Cancer Society recommends that doctors and dentists examine the mouth and throat during routine examinations [2] as early cancer lesions are often asymptomatic and may mimic benign lesions [19,20]. General population screening, however, has not been shown to reduce the incidence of and mortality from oral cancer. The reasons include the low prevalence and incidence of OSCC, the potential for false-positive diagnoses and poor compliance with screening and referral [6,21]. Thus the National Institute of Dental and Craniofacial Research and The Oral Cancer Foundation have recommended that research efforts focus on developing novel detection techniques [5,16].

Studies have reported that certain common oral bacteria are elevated on or in oral and esophageal cancer lesions and their associated lymph nodes [22-28]. Although increased colonization of facultative oral streptococci have been reported most often [24-27], anaerobic *Prevotella*, *Veillonella*, *Porphyromonas *and *Capnocytophaga *species were also elevated [25,26,28]. Currently, studies are examining whether bacteria may be incidentally or causally associated with oral cancer. Additional research is determining whether various salivary markers may be used as early diagnostic indicators for oral cancer.

The reason for these shifts in bacterial colonization of cancer lesions is unclear. Mechanistic studies of bacterial attachment provide some insights, however. Research has repeatedly shown that oral bacteria demonstrate specific tropisms toward different biological surfaces in the oral cavity such as the teeth, mucosa, and other bacteria [29-35]. The non-shedding surfaces of the teeth offer a far different habitat than the continually shedding surfaces of the oral mucosa. Due to the repeated shedding of epithelial cells, there is less time for a complex biofilm to develop on soft tissue surfaces; thus, a premium is placed on potent mechanisms of adhesion. The differences in bacterial tropisms for specific oral sites suggest that different intra-oral surfaces and bacterial species have different receptors and adhesion molecules that dictate the colonization of different oral surfaces.

It is now recognized that bacteria bind to and colonize mucosal surfaces in a highly selective manner via a "lock- and key" mechanism. Adhesins on bacteria bind specifically to complementary receptors on the mucosal surfaces of the host. These adhesins differ from species to species leading to specificity in attachment to different surfaces. Studies have shown that even within genera, colonization patterns of individual species may differ markedly [29-32]. *Streptococcus salivarius*, for example, preferentially colonized the oral soft tissues and saliva compared to the teeth, while the reverse was true of *Streptococcus sanguis*.

Cancer has been referred to as a molecular disease of cell membrane glycoconjugates, [36-38]. Certain glycoconjugates serve as receptors for specific bacteria and recent reports support the notion that shifts in the colonization of different cancer cells are associated with observed changes in cell surface receptors [36,40,41]. An *in vitro *study of *S. sanguis*, a common oral inhabitant, demonstrated that its binding capacity to normal exfoliated human buccal epithelial cells (HBEC) depended upon the availability of surface sialic acid residues [36]. Desialylation of HBEC invariably abolished adhesion of *S. sanguis *to these epithelial cells. In similar experiments carried out with a buccal carcinoma cell line, *S. sanguis *did not reliably attach. It was determined that the tumor cells did not express the sialylated membrane glycoprotein of normal cells suggesting that changes in the surface receptors had occurred in the buccal carcinoma cell line.

In a previous study of 225 OSCC-free subjects we found a high degree of specificity in the "preferred" intra-oral localization of species, even within a single genus such as *Streptococcus *[42]. This specificity in localization of individual species agreed with that described in previous studies. Our investigation extended earlier findings by describing the distribution of multiple species within the same genus on a wider range of intra-oral surfaces. For example, *S. oralis*, *S. constellatus*, *S. mitis*, *S. intermedius *and *S. anginosus *colonized the soft tissues in higher proportions than the teeth; however, their "preferred" soft tissue habitats differed. *S. sanguis *colonized different soft tissue locations in similar proportions, but was found in higher mean proportions on the teeth, particularly in the supragingival plaque.

The availability of a large amount of data from the OSCC-free subjects permitted this group to be subset according to periodontal and smoking status and the colonization patterns on the soft tissues compared among groups [43]. The clinical parameters among the populations were in accord with those found in previous studies and results were similar to previous investigations [44-47]. Few differences were found in the salivary or soft tissue microbiota among the subset populations. It was concluded that the presence or absence of periodontal infections or a smoking habit had minimal effects on salivary and soft tissue colonization. These findings were in accord with studies by Danser et al. 1996 and Lie et al. 1998 [48,49] but contrasted with earlier reports by Colman et al. 1976 and van Winkelhoff et al. 1986 [50,51]. Importantly, we found that when the microbiota of teeth, soft tissues and saliva were compared, the microbial profile of saliva was similar to that of the soft tissues, but saliva and soft tissue colonization differed markedly from that of dental plaque. These findings were similar to those of other investigations [46,51,52].

As previously mentioned, studies have reported that the microbiota of OSCC lesions differs from that found on the soft tissues of OSCC-free individuals. Little was known, however, about the salivary microbiota of oral cancer subjects. Thus, the purpose of the present investigation was to determine whether the salivary microbiota in subjects with an oral squamous cell carcinoma (OSCC) lesion would differ from that found in OSCC-free controls.

## Materials and methods

### OSCC-free Population

A total of 229 OSCC-free subjects were recruited from the patient pool at The Forsyth Institute. All subjects were 18 years or older, and immunocompetent. Exclusion criteria included: antibiotic therapy within the previous 3 months, pregnancy or lactation, systemic conditions associated with immune dysfunction (e.g., diabetes), previous chemotherapy or radiation and the presence of any oral mucosal lesions.

### Oral Cancer Population

A total of 45 subjects diagnosed with OSCC via biopsy were recruited from the Partners' Hospitals (The Dana Farber Cancer Institute, Brigham and Women's Hospital and Massachusetts General Hospital). Inclusion criteria required that subjects be 18 years or older and immunocompetent, with a primary untreated OSCC. Exclusion criteria included systemic conditions associated with immune dysfunction (e.g., diabetes), previous chemotherapy or radiation, an inability to properly consent, and/or lesions that could not be sampled due to discomfort, anatomic location or that did not affect the surface oral epithelium. In the unmatched comparisons OSCC subjects were older and included a higher percentage of male subjects and smokers than OSCC-free subjects (Table 1). Thus a subset of 45 controls was matched by computer for age, gender and smoking with the 45 OSCC subjects (Table 2).

**Table 1:** Age, gender and smoking status of 229 OSCC-free and 45 OSCC subjects.

	**OSCC-free**	**OSCC**
N	229	45
Mean Age (±SEM)	42.06 (±1.04)	57.6 (±2.34)
Minimum Age	18	18
Maximum Age	81	92
% Males (N)	47% (107)	71% (32)
% Smokers (N)	20% (46)	40% (18)

**Table 2:** Age, gender and smoking status of 45 OSCC-free and 45 OSCC subjects matched by age, gender and smoking history

	**OSCC-free**	**OSCC**
N	45	45
Age Mean (±SEM)	53.67 (±2.06)	54.46 (±2.27)
Age Min	19	18
Age Max	81	85
Males	32	32
Smokers	18	18

### Collection of samples and preparation of test membranes

Whole unstimulated saliva samples were collected by expectoration from 229 OSCC-free and 45 OSCC subjects. Samples were evaluated for their content of 40 common oral bacteria using checkerboard DNA-DNA hybridization as described by Socransky et al. [53] and whole genomic probes were prepared by the method described by Smith et al. (1989) [54]. The concentration of the purified DNA was determined by spectrophotometric measurement of the absorbance at 260 nm and purity of the preparations was assessed by the ratio of the absorbances at 260 and 280 nm. Whole genomic DNA probes were prepared from each of the 40 test strains by labeling 1–3 μg DNA with digoxigenin (Boehringer Mannheim, Indianapolis IN.) using a random primer technique [55]. The membranes were prehybridized to block nonspecific binding. The 40 species examined are commonly found in the oral cavity and are listed in Table 3 with their corresponding American Type Culture Collection (ATCC) numbers. Two lanes in each run had standards at 10^5 ^and 10^6 ^cells of each species and signals were converted to absolute counts by comparison with standards on the membrane. Signals were detected using a Storm Fluorimager (Molecular Dynamics, Sunnyvale CA). The sensitivity of this assay also detected 10^4 ^cells of a given species by adjusting the concentration of each DNA probe. Failure to detect a signal was recorded as zero, although counts in the 1 to 1000 range could have been present. Data available for all subjects were compared using the Mann-Whitney test and Bonferroni adjustment performed for multiple comparisons.

**Table 3:** The 40 test strains employed for the development of DNA probes.

**Microorganism**	**ATCC number**
*Actinobacillus actinomycetemcomitans*	43718 and 29523
*Actinomyces gerencseriae*	23860
*Actinomyces israelii*	12102
*Actinomyces naeslundii *genospecies 1	12104
*Actinomyces naeslundii *genospecies 2	43146
*Actinomyces odontolyticus*	17929
*Campylobacter gracilis*	33236
*Campylobacter rectus*	33238
*Campylobacter showae*	51146
*Capnocytophaga gingivalis*	33624
*Capnocytophaga ochracea*	33596
*Capnocytophaga sputigena*	33612
*Eikenella corrodens*	23834
*Eubacterium nodatum*	33099
*Eubacterium saburreum*	33271
*Fusobacterium nucleatum *ss *nucleatum*	25586
*Fusobacterium nucleatum *ss *polymorphum*	10953
*Fusobacterium nucleatum *ss *vincentii*	49256
*Fusobacterium periodonticum*	33693
*Gemella morbillorum*	27824
*Leptotrichia buccalis*	14201
*Neisseria mucosa*	19696
*Peptostreptococcus micros*	33270
*Porphyromonas gingivalis*	33277
*Prevotella intermedia*	25611
*Prevotella melaninogenica*	25845
*Prevotella nigrescens*	33563
*Propionibacterium acnes*	11827 and 11828
*Selenomonas noxia*	43541
*Streptococcus anginosus*	33397
*Streptococcus constellatus*	27823
*Streptococcus gordonii*	10558
*Streptococcus intermedius*	27335
*Streptococcus mitis*	49456
*Streptococcus oralis*	35037
*Streptococcus sanguis*	10556
*Tannerella forsythensis*	43037
*Treponema denticola*	B1
*Treponema socranskii*	S1
*Veillonella parvula*	10790

## Results

### Unmatched subjects

**Figure 1:**
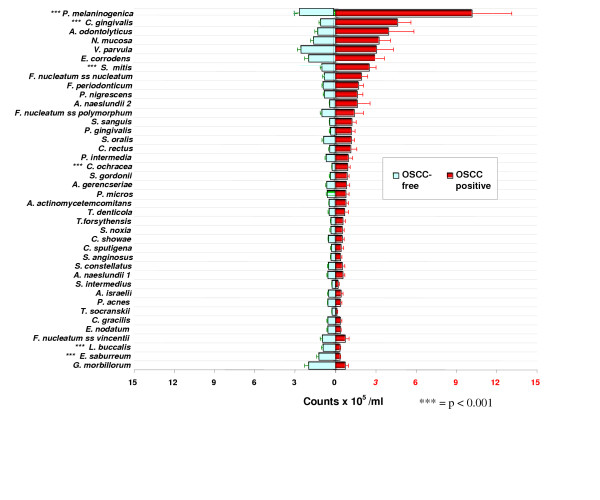
**Salivary counts for the 40 test species in both populations**. Mean counts (±SD) of 40 test species in saliva samples of 229 OSCC-free & 45 OSCC subjects (*** = p < 0.001).

The levels of salivary bacteria in subjects with and without OSCC are illustrated in Fig 1. Comparisons between the 229 OSCC-free and 45 OSCC subjects indicated that 6 common oral bacteria (*P. melaninogenica*, *C. gingivalis*, *Capnocytophaga ochracea*, *Eubacterium saburreum*, *Leptotrichia buccalis *and *S. mitis*) differed (*p *< 0.001). Elevated salivary counts of ≥0.4 × 10^5^/ml of 3 bacteria, *C. gingivalis*, *P. melaninogenica *and *S mitis*, were found to have diagnostic sensitivity and specificity ≥80%. The remaining species, including the three bacteria that were recovered in lower levels in salivary samples of OSCC patients, *E. saburreum*, *L. buccalis*, and *C. ochracea*, did not contribute significantly to the diagnostic test as their diagnostic sensitivity and specificity values were ≤60%.

**Figure 2:**
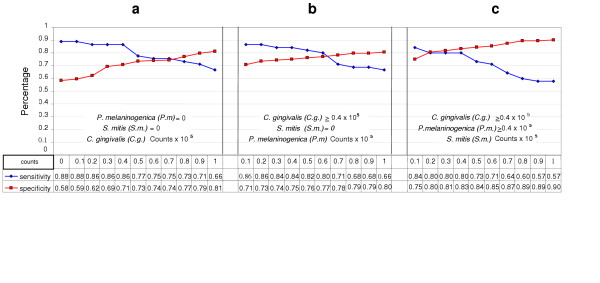
**Diagnostic sensitivity and specificity of 3 bacterial species in the *unmatched *populations**. **a**. Diagnostic sensitivity and specificity when *C. gingivalis *is at different salivary counts × 10^5^/ml and both *P. melaninogenica *and *S. mitis *= 0 **b**. Diagnostic sensitivity and specificity when *P. melaninogenica *is at different salivary counts × 10^5^/ml, *C. gingivalis *≥ 0.4 × 10^5^/ml and *S. mitis *= 0 **c**. Diagnostic sensitivity and specificity when *S. mitis *is at different salivary counts × 10^5^/ml and both *C. gingivalis *and *P. melaninogenica *≥0.4 × 10^5^/ml

Diagnostic sensitivity and specificity for different counts of *C. gingivalis*, *P. melaninogenica *and *S. mitis*/ml saliva for the unmatched populations are illustrated in Figure 2. *C. gingivalis *was the species most closely associated with oral cancer lesions but diagnostic sensitivity and specificity was highest when levels of the 3 bacteria were each ≥0.4 × 10^5^. Median DNA probe counts of *C. gingivalis P. melaninogenica *and *S. mitis *in OSCC-free subjects were 0.25, 0.63, and 0.31 × 10^5 ^per ml of saliva. In contrast, the median DNA probe counts of these 3 species in the 45 OSCC subjects were 3.24, 5.62 and 1.62 × 10^5 ^per ml of saliva, respectively.

### Matched Subjects

**Figure 3:**
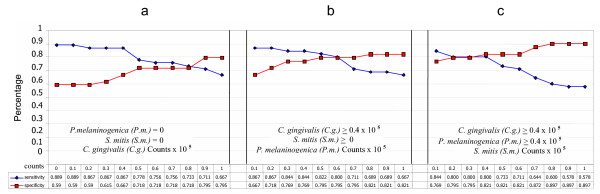
**Diagnostic sensitivity and specificity of 3 bacterial species in the *matched *populations**. **a**. Diagnostic sensitivity and specificity when *C. gingivalis *is at different salivary counts × 10^5^/ml and both *P. melaninogenica *and *S. mitis *= 0 **b**. Diagnostic sensitivity and specificity when *P. melaninogenica *is at different salivary counts × 10^5^/ml, *C. gingivalis *≥0.4 × 10^5^/ml and *S. mitis *= 0 **c**. Diagnostic sensitivity and specificity when *S. mitis *is at different salivary counts × 10^5^/ml and both *C. gingivalis *and *P. melaninogenica *≥0.4 × 10^5^/ml

45 OSCC-free controls were matched by computer for age, gender and smoking history with the 45 OSCC subjects. Diagnostic sensitivity and specificity for detection of oral cancer using levels of salivary bacteria were computed as described above. The results for the matched population were similar to those for the unmatched comparisons namely that the increased counts of *C. gingivalis*, *P. melaninogenica *and *S. mitis *were 80% diagnostically sensitive and 82% diagnostically specific for the presence of OSCC (Figure 3). As before, the remaining 37 species did not improve sensitivity or specificity.

## Discussion

Results from this investigation demonstrated that oral cancer subjects had elevated counts (p < 0.001) of *C. gingivalis*, *P. melaninogenica *and *S. mitis *in saliva compared to OSCC-free subjects. These results are borderline in significance after adjusting for multiple comparisons. However, when each species was ≥0.4 × 10^5 ^they indicated the presence of an OSCC lesion with 80% diagnostic sensitivity and ≥82% specificity in both matched and unmatched populations.

The reason for this finding is unclear. One explanation may relate to the altered cell surface receptors observed in cancer cells [36,39,41]. It seems reasonable that alterations in tumor cell receptors could change the adhesion of certain species of bacteria. This was shown, as previously discussed, in an *in vitro *study of HBEC and buccal cell carcinoma cell lines using the common oral bacterium *S. sanguis *by Neeser [36]. One might expect that as Neeser found decreased colonization of *S. sanguis*, a similar study using *S. mitis *would result in a reduced colonization of oral cancer cells. Interestingly, our previous investigation of 225 OSCC-free subjects provided evidence to the contrary. Colonization of different oral sites differed among the 40 test species, even among those of the same genera, such as streptococci. For example, *S. sanguis *and *S. mitis *both colonized the oral soft and hard tissues; however, marked differences in their proportions at these sites were noted. Highly species-specific oral colonization by streptococci has been reported by other investigators [29-32,57,58].

Saliva was found to be similar in microbial profile to the soft tissues. This was a significant finding from the study of the OSCC-free population. In contrast, the microbiota of the teeth and saliva differed markedly. These results agreed with previous studies [30,57,58]. Thus, if alterations in bacterial adhesion to OSCC cells observed *in vitro *exist *in vivo*, colonization of OSCC lesions would be affected. Shifts in the soft tissue microbiota of the oral cavity appear likely to affect salivary levels as well.

A screening test for oral cancer based on salivary counts of bacterial species is appealing. Saliva is now meeting the demand for inexpensive, noninvasive, and easy-to use diagnostic aids for oral and systemic diseases, and for assessing risk behaviors such as tobacco and alcohol use. Detection of HIV by the presence of virus-specific antibodies in saliva, for example, has led to the development of commercially available test kits [16]. If increased numbers of certain salivary species are shown to be a signature of oral cancer, an early diagnostic test for OSCC may be developed, reducing the morbidity and mortality of this devastating cancer.

Studies to examine the validity of these findings are planned. If the results of this study are validated it will be important to address whether oral bacteria can be used as indicators of oral cancer and whether certain oral species contribute to carcinogenesis.

## Conclusion

Results of the present study suggest that high salivary counts of *C. gingivalis*, *P. melaninogenica *and *S. mitis *may be diagnostic indicators of OSCC. These findings taken with those of an earlier study indicate that the presence of an OSCC has a more powerful effect on the salivary microbiota than either smoking or periodontal infections.

## Authors' contributions

DLM conceived of this investigation and the preliminary studies, constructed the study design, developed clinical sampling techniques, wrote hospital protocols and K-23 grant application that funded the project, collected and processed the majority of OSCC subject samples, modified laboratory protocols and drafted the manuscript. ADH made substantial contributions to the conception and study design. She coordinated the study of OSCC-free subjects, conducted the statistical analysis of the OSCC-free data and made substantial contributions to the interpretation of the data for the OSCC-free and OSCC populations. PMD, CMN and MRP made substantial contributions to the conception, design and coordination of the study during the preliminary studies and initiation of this investigation and were instrumental in coordinating clinical evaluations with the recruitment and sampling of OSCC subjects. JMG was instrumental in the conception and design of the study and performed the majority of statistical analyses and interpretations of data for the OSCC subjects. JMG made critical revisions for important intellectual content of the manuscript. All authors have given final approval of the manuscript.
